# Purification and production of *Plasmodium falciparum* zygotes from in vitro culture using magnetic column and Percoll density gradient

**DOI:** 10.1186/s12936-020-03237-1

**Published:** 2020-05-25

**Authors:** Yaxian Zhou, Alexis M. Grieser, Julie Do, Leslie S. Itsara, Ashley M. Vaughan, Anil K. Ghosh

**Affiliations:** 1 MalarVx, Inc, 1616 Eastlake Ave, E. Suite 285, Seattle, WA 98102 USA; 2 Seattle Children’s Research Institute, 307 Westlake Ave, N., Suite 500, Seattle, WA 98109 USA

**Keywords:** Malaria, *Plasmodium falciparum*, In vitro culture, Zygote purification, Ookinete transformation

## Abstract

**Background:**

*Plasmodium falciparum* zygotes develop in the mosquito midgut after an infectious blood meal containing mature male and female gametocytes. Studies of mosquito-produced *P. falciparum* zygotes to elucidate their biology and development have been hampered by high levels of contaminating mosquito proteins and macromolecules present in zygote preparations. Thus, no zygote-specific surface markers have been identified to date. Here, a methodology is developed to obtain large quantities of highly purified zygotes using in vitro culture, including purification methods that include magnetic column cell separation (MACS) followed by Percoll density gradient centrifugation. This straightforward and effective approach provides ample material for studies to enhance understanding of zygote biology and identify novel zygote surface marker candidates that can be tested as transmission blocking vaccine (TBV) candidates.

**Methods:**

*Plasmodium falciparum* gametocyte cultures were established and maintained from asexual cultures. Gametocytes were matured for 14 days, then transferred into zygote media for 6 h at 27 ± 2 °C to promote gamete formation and fertilization. Zygotes were then purified using a combination of MACS column separation and Percoll density gradient centrifugation. Purity of the zygotes was determined through morphological studies: the parasite body and nuclear diameter were measured, and zygotes were further transformed into ookinetes. Immunofluorescence assays (IFA) were also performed using the ookinete surface marker, Pfs28.

**Results:**

After stimulation, the culture consisted of transformed zygotes and a large number of uninfected red blood cells (RBCs), as well as infected RBCs with parasites at earlier developmental stages, including gametes, gametocytes, and asexual stages. The use of two MACS columns removed the vast majority of the RBCs and gametocytes. Subsequent use of two Percoll density gradients enabled isolation of a pure population of zygotes. These zygotes transformed into viable ookinetes that expressed Pfs28.

**Conclusion:**

The combined approach of using two MACS columns and two Percoll density gradients yielded zygotes with very high purity (45-fold enrichment and a pure population of zygotes [approximately 100%]) that was devoid of contamination by other parasite stages and uninfected RBCs. These enriched zygotes, free from earlier parasites stages and mosquito-derived macromolecules, can be used to further elucidate the biology and developmental processes of *Plasmodium.*

## Background

Malaria remains a major public health problem in sub-Saharan Africa. The recent global malaria report estimates the annual number of malaria deaths at 445,000, 90% of which occur in sub-Saharan Africa, followed by South-East Asia (7%) and the Eastern Mediterranean Region (2%) [1]. Of the nearly 250 *Plasmodium* species, only five species have been shown to infect humans–*Plasmodium falciparum*, *Plasmodium vivax*, *Plasmodium malariae*, *Plasmodium ovale*, and *Plasmodium knowlesi*. Of these, *P. falciparum*, the dominant species in Africa, accounts for the majority of deaths worldwide [2].

*Plasmodium falciparum* is carried by the *Anopheles* mosquito and the sporozoite stage is transmitted to humans by mosquito bite. Sporozoites migrate to the liver and invade hepatocytes, initiating liver stage development and giving rise to the production of thousands of merozoites through schizogony. Merozoites travel to the bloodstream, infect RBCs and undergo either asexual replication or differentiation into sexual precursor cells known as gametocytes. Sexual reproduction occurs in the mosquito midgut lumen when gametocytes are ingested by the mosquito in an infectious blood meal. Changes in the midgut microenvironment trigger the formation of female macrogametes and male microgametes. Following fertilization, gametes fuse and develop into diploid zygotes, which then undergo meiosis and transform into intermediate, immature ookinetes called retorts. Retorts matured into motile ookinetes transmigrate through the epithelial cells, settle beneath the basal lamina, and develop into mature oocysts after 10–14 days. Mature oocysts produce thousands of sporozoites that are released into the haemocoel and enter the salivary glands, where they are poised for transmission [3].

Strategies for eradicating malaria include medicines, vaccines, and vector control products, which target different stages of *Plasmodium* development. Transmission blocking vaccines (TBVs) represent a promising vaccine type that targets sexual stage antigens, thus interfering with the maturation and infectivity of the *Plasmodium* parasite inside the mosquito vector. TBVs impede the transmission of malaria infections among individuals by blocking infection of the mosquito. Current TBV candidates induce immune responses against the gametocyte and gamete surface antigens Pfs48/45 and Pfs230, as well as the zygote and ookinete surface antigens Pfs25 and Pfs28 [4–7]. Disrupting the function of Pfs48/45 and Pfs230 interrupts gamete fertilization, causing reduced zygote formation [8, 9]. Pfs25 and Pfs28 are responsible for ookinete survival and oocyst development, as demonstrated by a study in which loss of both Pfs25 and Pfs28 results in almost no oocyst formation in the mosquito midgut [10]. It has been shown that post-fertilization antigens can generate long-term immunity and develop fewer variations. The zygote is the first developmental stage after fertilization and is an essential intermediate between the haploid and diploid stages of the parasite. Discovery of novel antigens from the zygote surface may provide new candidates for TBV development.

The *P. falciparum* zygote has not been thoroughly studied, primarily due to the difficulty in extracting zygotes from the mosquito midgut and the resulting mosquito impurities remaining after zygote isolation. The development of an in vitro culture system provides a platform to produce *Plasmodium* mosquito stages in an environment free of mosquito debris. Several attempts have been made to purify zygotes, but none were successful in producing pure zygotes free from contamination by macrogametes and earlier parasite stages [11–13]. Thus, no methodology has been established for purification of *P. falciparum* zygotes free from other contaminating cell types. Here new culture and purification methods are described that combine magnetic isolation and a Percoll density gradient resulting in complete removal of RBCs and non-zygote parasites and yielding highly purified *P. falciparum* zygotes. This strategy enables production of pure material that can be used to further zygote studies and novel TBV development.

## Methods

### Reagents

Cell culture media and supplements were obtained from Corning Mediatech (Manassas, VA, USA). O^+^ human RBCs and human serum were obtained from Valley Biomedical (Winchester, VA, USA). Schneider’s media, Giemsa, DAPI, and Percoll were obtained from Sigma Aldrich (St. Louis, MO, USA). A MACS separator was obtained from Miltenyi (Bergisch Gladbach, Germany). Anti-Pfs25, and anti-Pfs28 antibodies were obtained from BEI Resources (Manassas, VA, USA). Alexa Fluor-conjugated anti-mouse IgG antibodies were obtained from Thermo Fisher Scientific [14].

### Blood stage culture

The GFP-expressing strain, *P. falciparum* NF54HT-GFP-*luc* [14] was maintained at 5% haematocrit in RPMI 1640 Media supplemented with 10% human serum, 2 mM l-glutamine, 25 mM HEPES, and 50 µM hypoxanthine at 37 °C under 5% CO_2_, 5% O_2_, 90% N_2_. Culture medium was replaced daily and new RBCs were added when the parasitaemia reached 3% [14, 15].

### Gametocyte culture

Gametocyte cultures were initiated from asexual parasites at 2% parasitaemia and 5% haematocrit. Cultures were maintained under the same conditions as described above for approximately 14 days with daily media changes without adding fresh RBCs. Exflagellation was studied from days 12 to 14 by counting exflagellation centers in 20 random and defined regions on a haemocytometer. The gametocyte culture was considered to be mature when more than one exflagellation event occurred in each region [16–18].

### Zygote induction

**Fig. 1:**
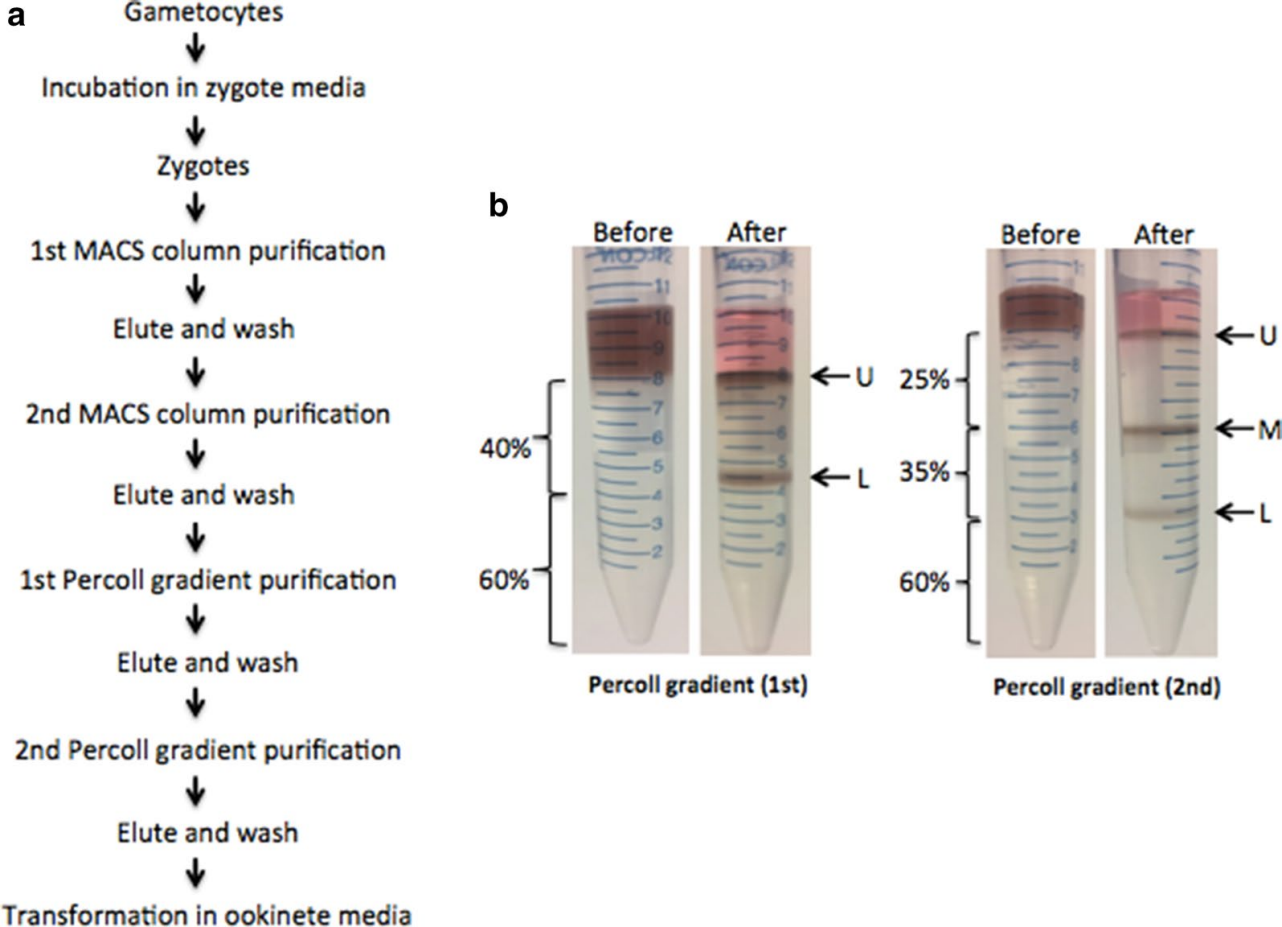
Purification method and zygote enrichment. **a** Flow diagram of zygote purification steps. **b** Percoll gradients, before and after centrifugation; bands indicated by arrows. *U* upper band, *M* middle band, *L* lower band

Mature gametocytes were obtained by centrifugation at 800×*g* for 10 min (9 min acceleration, 3 min brake) and resuspended in zygote media consisting of RPMI 1640, Schneider’s, and Waymouth’s media in equal portion with 20% fetal bovine serum (FBS), 4% human RBC lysate, 0.04% NaHCO_3_ (Sigma-Aldrich, MO), and 0.25% trehalose (Sigma-Aldrich, MO) at pH 7.4. Gametocytes were induced in zygote media for 6 h at 27 ± 2 °C to promote zygote transformation as described in the Fig. 1a legend [16].

### Purification of zygotes

#### Magnetic column purification

Transformed zygotes were concentrated and separated from infected RBCs by a magnetic column cell separation (MACS) system as previously described [19] with modification (Fig. 1a). Briefly, a Miltenyi LS column was placed into a Quadro MACS magnetic separator (Miltenyi Biotec, Bergisch Gladbach, Germany) and primed with 10 mL RPMI medium (incomplete, no serum) before adding parasite culture. Next, a 24-gauge blunted needle (Strategic Applications, IL) was attached to the end of the column. The zygote culture was transferred onto the column and the flow-through was collected and passed through a second primed column as described above. After washing with 10 mL RPMI medium, the column was detached from the magnetic separator and the zygotes were eluted with 5 mL RPMI 1640 media. An additional 5 mL of RPMI medium was added to the eluted solution before centrifuging at 1500 rpm for 8 min. The resulting pellet was resuspended in 5 mL RPMI 1640 medium for additional purification procedures.

#### Percoll gradient purification

MACS-sorted zygotes were further enriched by a two-step Percoll density gradient purification (two sequential density gradients). Purified zygotes were first suspended in 2 mL RPMI medium and layered on a discontinuous gradient consisting of 60% (bottom layer) and 40% (top layer) Percoll solutions (all Percoll was diluted in PBS). Next, the gradient was centrifuged at 1500x*g* (9 min acceleration, 3 min brake) for 15 min at room temperature (RT). The parasites within the band above the 40% Percoll layer were collected and washed once with PBS. The second gradient consisted of 60% (bottom), 35% (middle) and 25% (top) Percoll solutions. Parasites collected from the prior step were loaded on the top of the gradient and centrifuged as described above. The parasite bands located at the interfaces of the (−25% Percoll, 25% Percoll–35% Percoll, and 35% Percoll–60% Percoll) were washed with PBS (Fig. 1b). Each group of parasites collected was individually incubated in ookinete media for ookinete transformation. This method of purification using two MACS columns followed by two Percoll gradient centrifugation steps is hereafter referred to as “2 MACS 2 Percoll”.

Without MACS purification, the transformed zygotes were directly layered on the gradient consisting of 60% (bottom layer), 35% (middle layer), and 25% (top layer) Percoll solutions. The gradient was centrifuged at 1000x*g* for 30 min. This method of purification using only one Percoll gradient centrifugation step is hereafter referred to as “Percoll only”. After one MACS column purification, sorted zygotes were loaded onto the top of the gradient consisting of 60% (bottom), 35% (middle) and 25% (top) Percoll or Accudenz solutions. The gradient was centrifuged at 1000x*g* for 30 min. This method of purification using one MACS column followed by one Percoll gradient or one Accudenz gradient is hereafter referred to as “1 MACS 1 Percoll” or “1 MACS 1 Accudenz”.

### Ookinete transformation

To induce ookinete transformation, purified parasites from the three interfaces were washed in PBS, transferred to a 12-well plate, and incubated in 200 µL of ookinete medium (identical to zygote medium) at 27 ± 2 °C. Ookinete transformation was studied over a time course of 24 h. Specific parasite morphology was studied at 0, 8, 16, and 24 h post-incubation. Fifty µl of culture was collected from each well for immunofluorescent staining and smeared on a standard microscope slide [18].

### Counting and identification of different parasite stages

RBCs, gametocytes, macrogametes, and zygotes were collected from 10 ml of zygote culture and counted under a haemocytometer. Ookinetes were obtained from 200 µl of ookinete culture. Different stages were identified according to their shape and size. RBCs are cells with round and reddish color, Gametocytes are elongated, and ookinetes are elongated banana shaped. Zygotes are oval shaped, while macrogametes are circular and transparent. Two experiments were performed and the representative data was shown. Data is presented in tables as total cells counted.

### Giemsa staining

Before and after each step of purification, 10 µl of culture was collected and smeared on a slide. Each slide was fixed with methanol and stained with 10% Giemsa solution in water for 10 min before imaging.

### Immunofluorescence assays

Samples collected after each purification step during the ookinete- induction time course were fixed on glass slides using 4% paraformaldehyde in PBS for 15 min. After washing with PBS, the slides were blocked in blocking buffer (3% BSA in PBS) for 1h and incubated with primary antibodies against Pfs25 (MRA-28, 1:300) or Pfs28 (MRA-17, 1:300) for 1h at RT. Slides were then washed three times and blocked again as described above. Next, a secondary antibody (Alexa Fluor 594, Goat Anti-Rat IgG, 1:1000) was added and incubated for 45 min at RT before mounting with slowfade-antifade mounting medium containing DAPI (Thermo Fisher Science, MA) [18].

### Macrogamete purification and flow cytometry

Zygotes were purified as described above through two MACS columns and two Percoll density gradients. Macrogametes were collected from the flow-through of the two MACS columns and further purified with a Nycodenz gradient [20]. After purification, both zygotes and macrogametes were fixed with 4% para-formaldehyde in PBS for 15 min and permeabilized for 5 min with 0.1% TritonX-100 in PBS. After being washed with PBS, test samples were incubated with Vybrant DyeCycle Orange DNA stain (Thermo Fisher Scientific Inc., MA) at a concentration of 10 uM for 30 min at 37 °C and washed again. Samples were resuspended in 2% BSA/2 mM EDTA in PBS. Before analysis, samples were filtered into polystyrene tubes through mesh cell-strainer caps. Flow cytometry for both macrogametes and zygotes was performed on either a BD LSR II or BD FACSymphony flow cytometer (BD Biosciences, CA). Analysis was conducted using FlowJo software (FlowJo, version 10.6.1).

### Macrogamete and zygote differentiation

Gametocytes were incubated in zygote media for 15 or 30 min and smeared on glass slides. Parasites were fixed with either methanol or paraformaldehyde as describe above. Methanol-fixed slides were stained with Giemsa. Paraformaldehyde-fixed slides were stained with anti-Pfs25 as described above. The nuclear diameter of zygotes and macrogametes was measured by using a micrometer on Giemsa-stained slides with a light microscope or by using measurement software on DAPI-stained slides with a confocal microscope [21].

### Microscopy

Giemsa-stained images were acquired with a light microscope (Nikon Eclipse 50i). Images were viewed at 40x, 60x, or 100x magnification with a 10x eyepiece. All other images were taken using a DeltaVision Elite High Resolution Microscope (GE Healthcare Life Science, PA) designed for fluorescence imaging and analysed using DeltaVision software (SoftWoRx software, version 6.5.2).

## Results

### Confirmation of zygotes

**Fig. 2:**
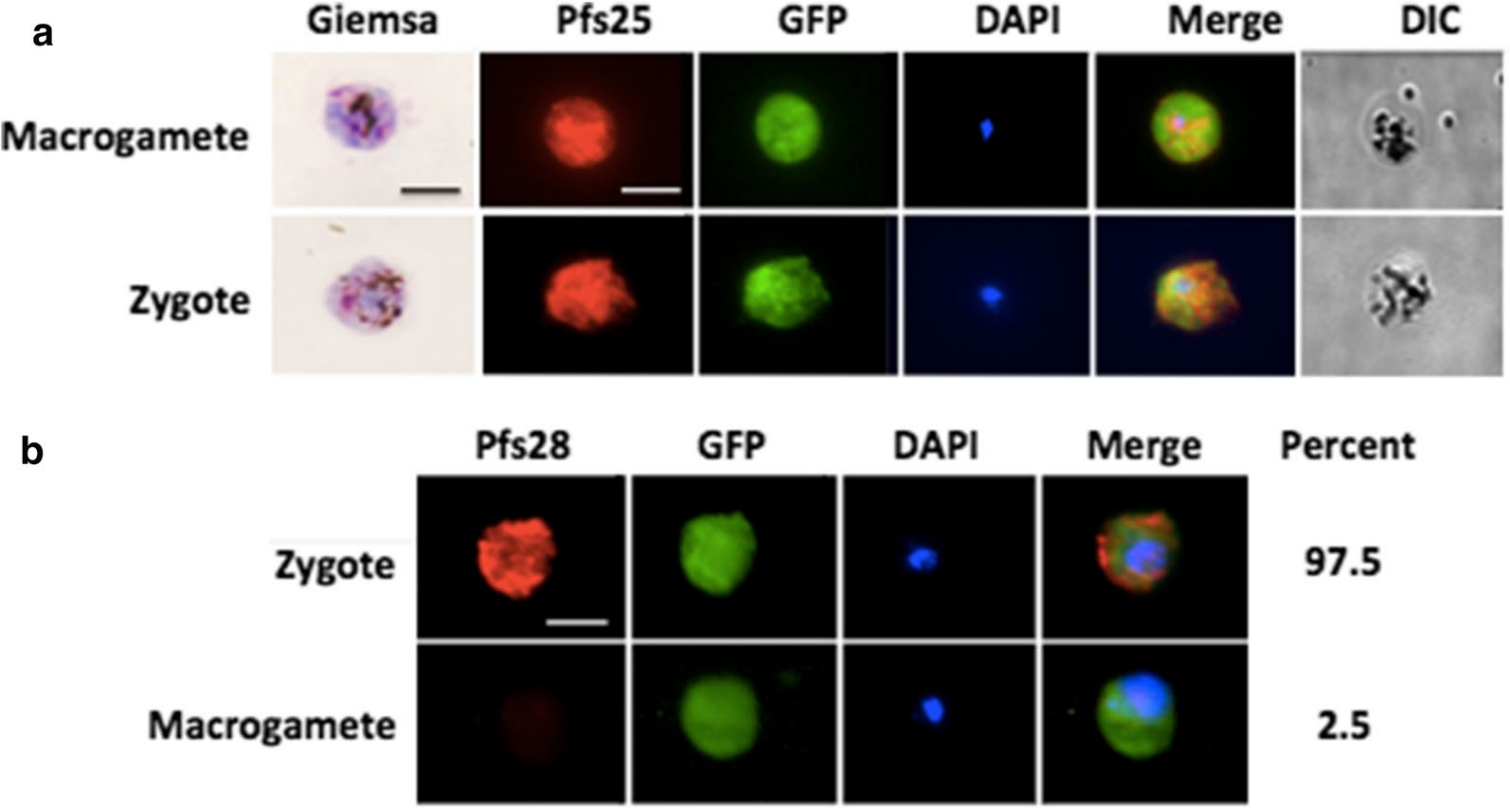
Comparison between zygotes and macrogametes. Zygotes were purified by 2 MACS and 2 Percoll methods and macrogametes were purified from the flow-through of the two MACS columns and further purified with a Nycodenz gradient. **a** Giemsa and Pfs25 staining. **b** Pfs28 staining. The percentage of cells expressing Pfs28 is presented

Zygotes and macrogametes are similar in size. Most previously published purification methods were not able to differentiate them [11]. To distinguish zygotes from macrogametes, body and nuclear size were compared through Giemsa and IFA staining. Zygotes were purified by 2 MACS and 2 Percoll methods and macrogametes were purified from the flow-through of the two MACS columns and further purified with a Nycodenz gradient [20]. Fixed parasites were stained with an anti-Pfs25 antibody and DAPI (for nuclear staining) (Fig. 2a). The zygotes were verified by Pfs28 staining (Fig. 2b). While body diameter is comparable between zygotes and macrogametes, zygotes display significantly larger nuclei (Table 1), in agreement with previous observations [19]. The difference in nuclear diameter served as a metric to distinguish zygotes from macrogametes during the purification experiments. The DNA content of the zygote is more than that of the macrogametes (Additional file 1: Figure S1).

**Table 1:** Body and nuclear size of macrogametes and zygotes

	Giemsa		IFA/DIC	
	Cell body (µ)	Nuclear (µ)	Cell body (µ)	Nuclear (µ)
Mac Gam	6.42 ± 0.47	0.73 ± 0.07	6.12 ± 0.56	0.83 ± 0.12
Range	(6.0–7.0)	(0.6–0.8)	(5.0–7.6)	(0.7–1.0)
Zygote	6.87 ± 0.25	1.86 ± 0.20	6.69 ± 0.82	1.23 ± 0.20
Range	(6.5–7.0)	(1.4–2.2)	(5.0–8.2)	(0.7–1.9)

The cell body size and the nuclear size of macrogametes and zygotes are shown in the table as mean ± SD. The ranges of the size are also given

### MACS columns removed the majority of RBCs and gametocytes

Gametocytes matured after 6 h of incubation in the zygote medium and transformed into zygotes. At this point, the parasite population consisted of zygotes, along with a large number of uninfected RBCs, untransformed gametocytes, and macrogametes (Table 2). Two sequential MACS columns were used for zygote purification. After the first MACS column, 93.8% of gametocytes, 78.1% of macrogametes, and 99.9% of uninfected RBCs had been removed. After the second column, 98.4% of gametocytes, 94.3% of macrogametes, and 100% percent of uninfected RBCs were removed compared to the starting parasite culture (Table 2). Thus, two sequential magnetic columns efficiently removed uninfected RBCs and a significant proportion of earlier parasite stages (gametocytes and macrogametes). Zygotes were enriched after using the first MACS column (30.9 fold) and were further enriched after using a second MACS column (37.7 fold). Further purification methods were tested to identify a protocol capable of yielding pure, highly enriched zygotes.

**Table 2:** Enrichment of zygotes during purification using “2 MACS 2 Percoll” method

Parasite/Cell	Before Purification (after 6 h in ookinete media)	After ^1st^ MACS column	After 2nd MACS column	After 1st Percoll column		After 2nd Percoll column		
				Upper band	Lower band	Upper band	Middle band	Lower band
RBC	6.0x10^9^	5.0x10^6^	0	0	0	0	0	0
Gam	35.3x10^7^	2.2x10^7^	5.8x10^6^	1.7x10^6^	2.1x10^6^	0	7.4x10^5^	2.6x10^5^
Zygote	14.4x10^7^	6.7x10^7^	3.4x10^7^	1.7x10^7^	4.8x10^6^	2.8x10^6^	6.0x10^6^	3.0x10^5^
Mac Gam	2.1x10^7^	4.6x10^6^	1.2x10^6^	2.0x10^5^	6.0x10^5^	0	1.5x10^5^	0
Zygote %	2.2%	67.9%	82.9%	89.9%	64.0%	100%	87.1%	53.5%

Absolute total numbers of RBCs and different parasite stages before and after each purification step are shown. Enrichment of zygote is indicated by the percentage of zygote in the whole population after each purification step

*RBC  *red blood cells, *Gam* gametocytes, *Mac Gam* macrogametocytes

### Percoll gradients enriched zygotes in distinct bands

A simpler method of zygote purification using an Accudenz (previously known as Nycodenz) gradient after MACS column purification was tested. A gradient of 60%, 35%, and 25% Accudenz [19] was centrifuged at low speed (1000x*g*) for 30 min. Centrifugation produced two bands; zygotes were only present in the upper band. However, the upper band was also contaminated with gametocytes and uninfected RBCs (upper band: 40.7% zygotes). Only cell debris and haemozoin pigment were found in the lower band (Additional file 2, 3: Figure S2a and Table S1). Another purification method was tested in which no MACS column was used and parasites were layered onto a gradient consisting of 60% (bottom layer), 35% (middle layer), and 25% (top layer) Percoll solutions. After centrifugation at 1000x*g* for 30 min, three bands were formed, but the entire gradient was smeared with lysed RBCs (Additional file 2: Figure S2b), likely due to RBCs bursting during centrifugation. Moreover, all three bands were contaminated with gametocytes (Additional file 4: Table S2). Thus the use of a single MACS column with Accudenz and a single Percoll gradient did not improve on zygote purification compared to two sequential MACS columns.

**Fig. 3:**
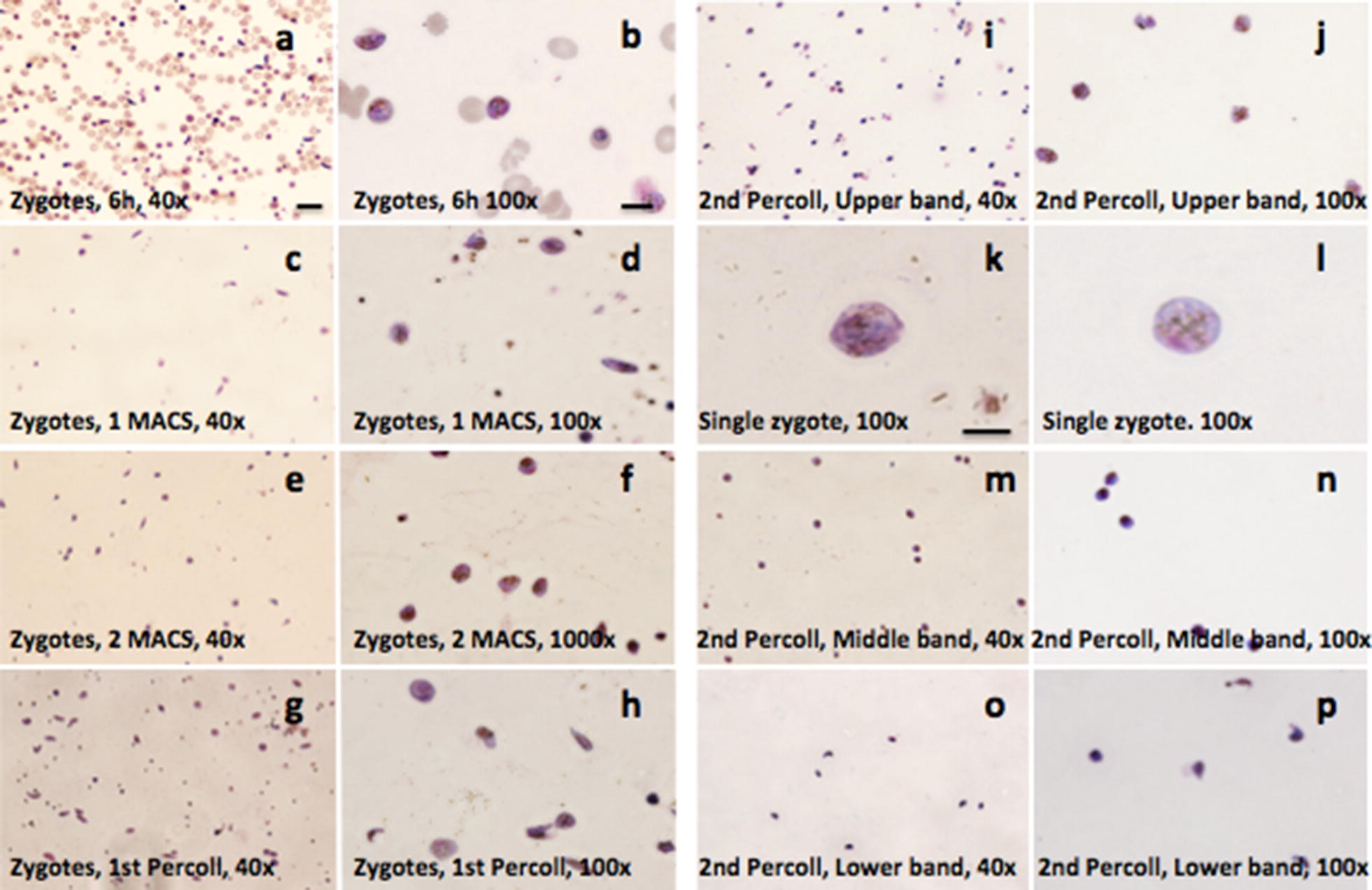
Giemsa staining of purified material from 2 MACS and 2 Percoll gradients. **a** Zygotes after 6 h in ookinete media at 40x; **b** Same at 100x; **c** Zygotes after 1st MACS at 40x; **d** Same at 100x; **e** Zygotes after 2nd MACS at 40x; **f** Same at 100x; **g** Zygotes after 1st Percoll at 40x; **h** Same at 100x; **i** Zygotes from 2nd Percoll upper band at 40x; **j** Same at 100x; **k** Same enlarged; **l** Same in different field; **m** Zygotes from 2nd Percoll middle band at 40x; **n** Same at 100x; **o** Zygotes from 2nd Percoll lower band at 40x; **p** Same at 100x. Panel a, c, e, g, i, m, o: scale bar = 20 µm; b, d, f, h, j, n, p: scale bar = 10 µm; k, l: scale bar = 5 µm

Since neither of these methods proved effective at removing earlier *P. falciparum* stages (gametocytes and macrogametes), two sequential discontinuous Percoll gradients were used after the two MACS columns. For the first Percoll gradient, 60% (bottom) and 40% (top) layers were used. Centrifugation gave rise to two separate bands containing parasites (Fig. 1b). The upper band consisted of a higher number of zygotes (1.7 x 10^7^) compared to the lower band (4.8x10^6^). To further purify the zygotes, the parasites in the upper band were added to a second Percoll gradient: 60% (bottom layer), 35% (middle layer), and 25% (top layer). Centrifugation yielded three separate bands containing parasites (Fig. 1b). The purest population of zygotes was present in the upper band (45.5-fold enrichment; 100% zygotes, compared to 2.2% zygotes before purification). The middle and lower bands contained fewer zygotes and were contaminated with RBCs, gametocytes, and macrogametes along with cell debris and haemozoin pigment. (Figure 3 and Table 2).

While the use of two MACS columns and two Percoll gradient centrifugation steps provides a pure population of zygotes, it also causes a moderate loss of transformed zygotes due to multiple steps of purification. To attempt to minimize the loss of zygotes during purification, the use of a single MACS column and a single Percoll density gradient (60%, 35%, and 25%) at a lower centrifugation speed (1000x*g*) and longer spinning time (30 min) was tested. After centrifugation, three bands consisting of parasites were present, and zygotes were present in all bands. However, there was greater contamination from other parasites stages (the final zygote percentage was 88.9%; Additional file 2, 5: Figure S2c and Table S3). Thus, the method “2 MACS 2 Percoll” is the preferred method to obtain the purest population of zygotes. The experiments of “2 MACS 2 Percoll” were performed in duplicates. Best results were presented in the text and similar results were obtained from the other experiments (Additional file 6: Table S4).

### Zygote to ookinete transformation

**Fig. 4:**
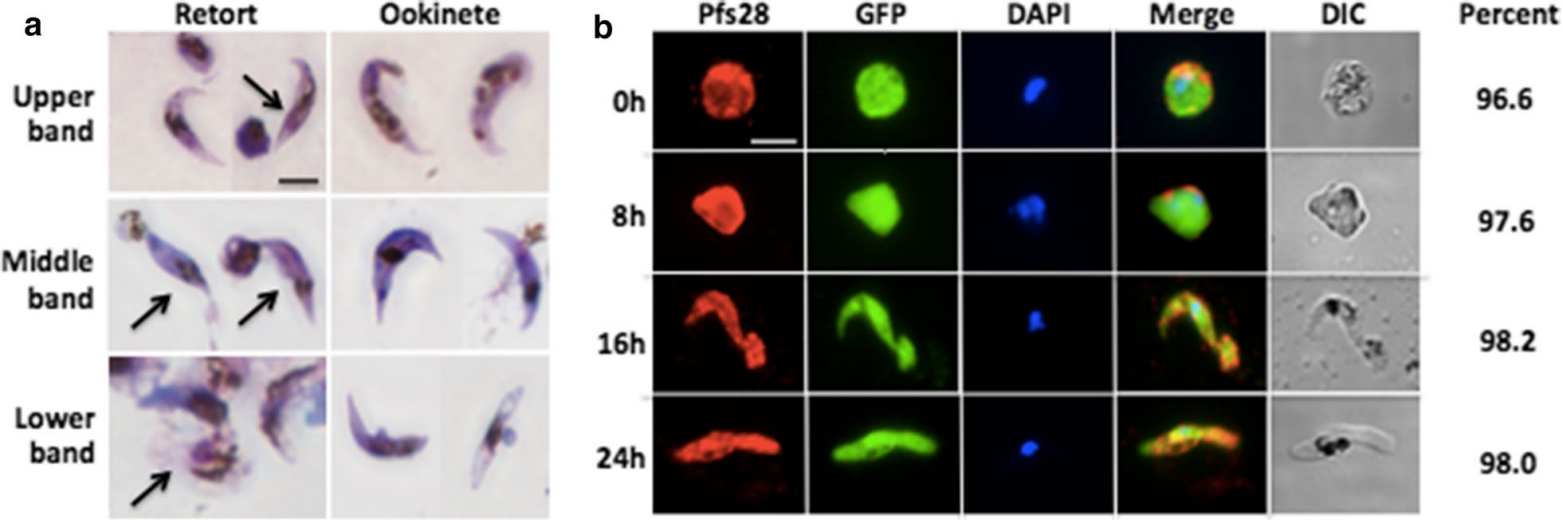
Expression of Pfs28 in zygotes and transformed ookinetes. **a** Giemsa staining of retorts and ookinetes after 2nd Percoll gradient. **b** IFA picture of zygotes from the upper Percoll band and transformed retort and ookinete at different time points, staining with anti-Pfs28 antibody. GFP was expressed to indicate the parasite. DAPI was used to indicate the nuclei. DIC was presented to show the cell shape. The percentages of cells expressing Pfs28 at the different times analysed during the 24 h time course were presented. Scale bar = 5 µm

Parasites separated via the 2 MACS 2 Percoll protocol were subjected to zygote-to-ookinete transformation in ookinete medium for an additional 24 h at 27 °C. A larger percentage of zygotes from the upper band transformed into ookinetes compared to the middle and lower bands (Table 3). The percent of transformation, indicated as P trans (z-o), was calculated as the ratio of the number of transformed ookinetes to the number of zygotes. Zygotes purified by 1 MACS 1 Accudenz and 1 MACS 1 Percoll methods were also incubated in ookinete media. The percentages of transformed ookinetes from these two methods were much lower compared to those measured after the 2 MACS 2 Percoll protocol (Additional file 7: Table S5). Purified zygotes were incubated in ookinete medium and sampled over a 24-h time course. Giemsa staining showed that purified zygotes from all three bands were able to transform into retorts and then ookinetes (Fig. 4a). Zygote, retort, and mature ookinete morphology was studied by IFA using Pfs28, a surface marker of these stages [10]. Early stage retorts with a small protrusion, as well as late stage retorts were found at 8 and 16 h of the transformation. Expression of Pfs28 confirmed the presence of zygotes in the purified samples. Transformed ookinetes were morphologically similar to mosquito-derived ookinetes and expressed Pfs28 (Fig. 4b).

**Table 3:** Transformation of zygotes into ookinetes after purification

Parasite/Cell	After 2nd Percoll column		
	Upper band	Middle band	Lower band
Zygote	2.8x10^6^	6.0x10^6^	3.0x10^5^
Ookinete	7.0x10^5^	9.0x10^5^	4.2x10^4^
P trans (z-o)	25.0%	15.0%	14.0%

Absolute total number of zygotes after 2 MACS 2 Percoll purification are shown. The number of ookinetes after transformation and percentage of zygotes that transformed into ookinetes are also given. P trans (z-o) = percent transformation, zygote to ookinete

## Discussion

### Previous zygote purification methods

Classical zygote purification methods utilize either wheat germ agglutinin (WGA) to gather RBCs away from parasites [13, 22] or lysis of RBCs with ammonium chloride [23]. WGA does not remove RBCs effectively due to differential binding of the WGA to young and old RBCs [24]. Ammonium chloride solution treatment also does not lyse RBCs completely. It needs further purification to remove the residual RBCs.

Other attempts have been made to isolate *Plasmodium* macrogametes and zygotes, including the use of metrizamide (a non-ionic radiopaque density gradient medium for the centrifugation of biological particles, now unavailable) to separate macrogametes and zygotes prepared from in vitro cultures: gametocytes were layered in a discontinuous gradient (70% and 10%) and centrifuged. Recovered parasites were further purified using a WGA-Sepharose 6 MB column and 67% of macrogametocytes were recovered [11]. A separate study to isolate *Plasmodium gallinaceum* zygotes used a Hypaque/Ficoll gradient method [13]. The interface material was collected and zygotes, macrogametes, white blood cells, and RBCs were found. Infected and uninfected RBCs were removed by several rounds of WGA agglutination processing followed by column purification through glass wool. The final sample contained zygotes (90% pure) with contaminating macrogametes.

A similar study using the NF54 strain (wild type) of *P. falciparum* employed an Acccudenz gradient to purify macrogametes and zygotes. Briefly, gametocytes were transformed into gametes and zygotes. A density gradient of Accudenz (16% lower, 11% middle, and 6% top layers) was made and the transformed material was loaded onto the gradient and centrifuged. Macrogametes and zygotes were harvested from the 6–11% interface. The percent purity was not determined and the samples were contaminated with RBCs [12]. The method described here (2 MACS 2 Percoll) improves on these earlier procedures, which were less effective in producing a pure zygote population.

### Advantages of MACS magnetic columns

The 2 MACS 2 Percoll method provides advantages over other methods because Percoll (a relatively low-viscosity colloidal silica that is regularly used in purification of various cells types) efficiently separates zygotes without causing physical or functional damage [25]. Removal of RBCs with a MACS column is a simple and quick method and has been used previously to separate other *Plasmodium* stages [13, 23, 26]. It was found that zygotes bind to the column and other cell types were removed from the columns during the washing steps.

The methodology described here produces large quantities of highly purified zygotes that could be used to identify new markers for further study. These potentially important zygote antigens could be targeted individually or in combination with known sexual stage antigens [27–30]. Additionally, these markers could be used to assess the quality of an in vitro system for the mass production of a genetically attenuated parasite (GAP) vaccine [31]. For example, sexual stage antigens could serve as developmental biomarkers and function as quality control reagents for culturing systems.

## Conclusion

Development of an easy in vitro culture and purification system is necessary for understanding the dynamics of parasites reproduction, gametocyte biology, and malaria transmission rates. Identification of novel proteins on the zygote surface could provide insight for malaria control. The method of zygote purification described here is a highly efficient and simple means to produce and purify *P. falciparum* zygotes from in vitro gametocyte culture. The combination of two MACS columns and two Percoll density gradients enables the purification of a large number of zygotes free of contaminating RBCs and other parasite stages. It is presumed that several stage-specific proteins are expressed only on the surface of zygotes, but no specific markers have been identified to date. To aid in the identification of stage-specific zygote markers, membrane isolation and mass spectrometry analysis from pure populations of *Plasmodium* zygotes should be performed. This may allow identification of novel surface markers for use in TBVs.

## Supplementary information

**Additional file 1: Figure S1.** Flow cytometry analysis on macrogametes and zygotes. Macrogamete populations were purified through MACS columns and Nycodenz gradients. Zygote populations were purified through MACS columns and Percoll gradients. 10,000 events were recorded for each sample. Control populations expressed GFP measured with a FITC-488 laser; test populations expressed both GFP and DyeCycle Orange DNA stain measured with Cy5.5 laser. The gating tree was set as follows: SSC-H vs. SSC-A (doublet exclusion); SSC-A vs. FSC-A (population of interest); GFP-A/Alexa Fluor 488-A vs. PE-Cy5-5-A (DNA content of GFP-expressing cells). **a** Representative histogram from one experiment displaying DNA content (PE-Cy5-5-A) through expression of Cy5.5 DNA stain, normalized to mode. Blue peak corresponds to macrogamete population, red peak corresponds to zygote population. **b** Chart displaying DNA content of zygote populations (darker bar) relative to macrogamete populations (lighter bar). Median PE-Cy5-5-A expression values from four separate experiments were averaged for macrogametes or zygotes and displayed as relative DNA content (n).

**Additional file 2: Figure S2.** Percoll gradients of other purification method. **a** Accudenz gradient after 1st MACS column purification. **b** Percoll gradient without using MACS purification. **c** Percoll gradient after 1 MACS column. *U* upper band, *M* middle band, *L* lower band.

**Additional file 3: Table S1.** Enrichment of zygotes during purification using “1 MACS 1 Accudenz” method.

**Additional file 4: Table S2.** Enrichment of zygotes during purification using “Percoll only” method.

**Additional file 5: Table S3.** Enrichment of zygotes during purification using “1 MACS 1 Percoll” method.

**Additional file 6: Table S4.** Enrichment of zygotes during purification using “2 MACS 2 Percoll” method.

**Additional file 7: Table S5.** Transformation of zygotes into ookinetes after purification.

## Data Availability

The dataset supporting the conclusion is available from the corresponding author upon request.

## Abbreviations

MACS: Magnetic column cell separation
RBCs: Red blood cells
TBVs: Transmission blocking vaccines
FBS: Fetal bovine serum
RPMI: Roswell Park Memorial Institute
RT: Room temperature
IFA: Immunofluorescence assays
WGA: Wheat germ agglutinin
GAP: Genetically attenuated parasite
