# Supplementary material for: Purification and production of Plasmodium falciparum zygotes from in vitro culture using magnetic column and Percoll density gradient
Source: Malar J. 2020 May 25;19:192. doi: 10.1186/s12936-020-03237-1 (PMC7249376; doi:10.1186/s12936-020-03237-1)
Supplement: Supplementary file 1 — Additional file 1: Figure S1. Flow cytometry analysis on macrogametes and zygotes. Macrogamete populations were purified through MACS columns and Nycodenz gradients. Zygote populations were purified through MACS columns and Percoll gradients. 10,000 events were recorded for each sample. Control populations expressed GFP measured with a FITC-488 laser; test populations expressed both GFP and DyeCycle Orange DNA stain measured with Cy5.5 laser. The gating tree was set as follows: SSC-H vs. SSC-A (doublet exclusion); SSC-A vs. FSC-A (population of interest); GFP-A/Alexa Fluor 488-A vs. PE-Cy5-5-A (DNA content of GFP-expressing cells). a Representative histogram from one experiment displaying DNA content (PE-Cy5-5-A) through expression of Cy5.5 DNA stain, normalized to mode. Blue peak corresponds to macrogamete population, red peak corresponds to zygote population. b Chart displaying DNA content of zygote populations (darker bar) relative to macrogamete populations (lighter bar). Median PE-Cy5-5-A expression values from four separate experiments were averaged for macrogametes or zygotes and displayed as relative DNA content (n). [file 12936_2020_3237_MOESM1_ESM.pdf]

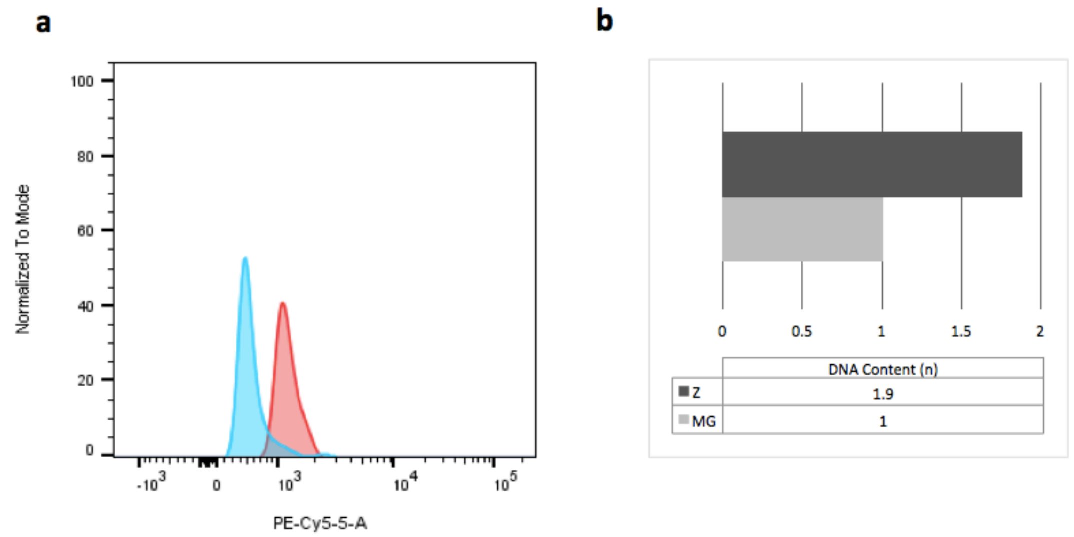

Fig. S1

Figure S1. Flow cytometry analysis on macrogametes and zygotes. Macrogamete populations were purified through MACS columns and Nycodenz gradients. Zygote populations were purified through MACS columns and Percoll gradients. 10,000 events were recorded for each sample. Control populations expressed GFP measured with a FITC-488 laser; test populations expressed both GFP and DyeCycle Orange DNA stain measured with Cy5.5 laser. The gating tree was set as follows: SSC-H vs. SSC-A (doublet exclusion); SSC-A vs. FSC-A (population of interest); GFP-A/Alexa Fluor 488-A vs. PE-Cy5-5-A (DNA content of GFP-expressing cells). **a** Representative histogram from one experiment displaying DNA content (PE-Cy5-5-A) through expression of Cy5.5 DNA stain, normalized to mode. Blue peak corresponds to macrogamete population, red peak corresponds to zygote population. **b** Chart displaying DNA content of zygote populations (darker bar) relative to macrogamete populations (lighter bar). Median PE-Cy5-5-A expression values from four separate experiments were averaged for macrogametes or zygotes and displayed as relative DNA content (n).
