# Supplementary material for: Purification and production of Plasmodium falciparum zygotes from in vitro culture using magnetic column and Percoll density gradient
Source: Malar J. 2020 May 25;19:192. doi: 10.1186/s12936-020-03237-1 (PMC7249376; doi:10.1186/s12936-020-03237-1)
Supplement: Supplementary file 2 — Additional file 2: Figure S2. Percoll gradients of other purification method. a Accudenz gradient after 1st MACS column purification. b Percoll gradient without using MACS purification. c Percoll gradient after 1 MACS column. U upper band, M middle band, L lower band. [file 12936_2020_3237_MOESM2_ESM.pdf]

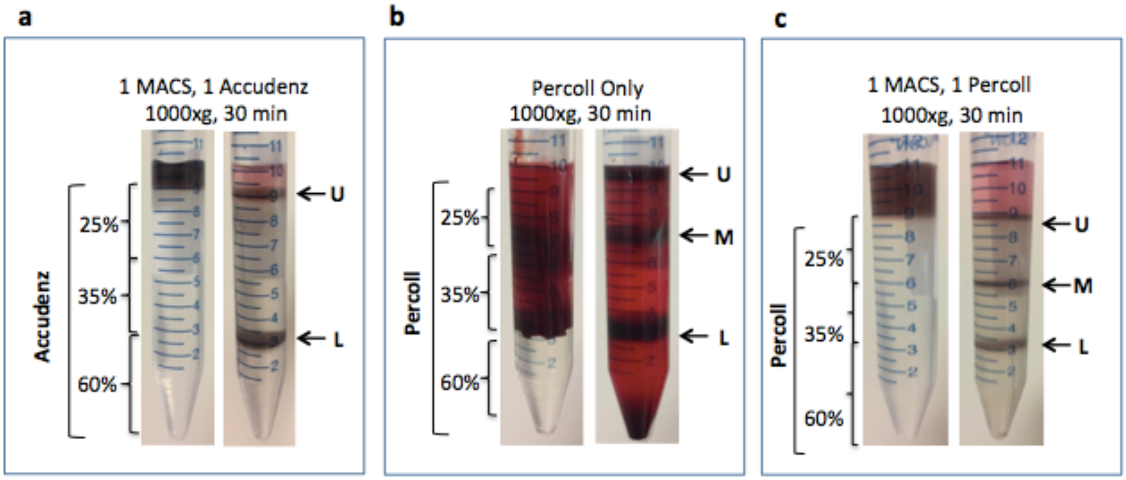

Figure S2. Percoll gradients of other purification method. **a** Accudenz gradient after 1st MACS column purification. **b** Percoll gradient without using MACS purification. **c** Percoll gradient after 1 MACS column. *U* upper band, *M* middle band, *L* lower band.
