# Supplementary material for: Purification and production of Plasmodium falciparum zygotes from in vitro culture using magnetic column and Percoll density gradient
Source: Malar J. 2020 May 25;19:192. doi: 10.1186/s12936-020-03237-1 (PMC7249376; doi:10.1186/s12936-020-03237-1)
Supplement: Supplementary file 5 — Additional file 5: Table S3. Enrichment of zygotes during purification using “1 MACS 1 Percoll” method. [file 12936_2020_3237_MOESM5_ESM.pdf]

Table S3: Enrichment of zygotes during purification using "1 MACS 1 Percoll" method

Parasite/Cell	Before Purification	After Column	Percoll, 1000xg, 30 min		
			upper band	middle band	lower band
RBC	9.0x10^9	4.0x10^6	0	0	2.0x10^5
Gam	1.5x10^8	1.2x10^7	1.0x10^5	5.0x10^5	2.7x10^6
Zygote	2.4x10^8	1.8x10^7	4.0x10^6	4.2x 10^6	2.1x10^6
Mac Gam	1.4 x10^8	4.8x10^6	4.0x10^5	3.0x10^5	6.0x10^5
Zygote %	2.5%	46.4%	88.9%	84.0%	37.5%

Absolute total numbers of RBCs and different parasite stages before and after each purification step are shown. Enrichment of zygotes is indicated by the percentage of zygotes in the whole population after each purification step (Zygote %). RBC = red blood cells; Gam = gametocytes; Mac Gam = macrogametocytes.
