# Supplementary material for: Purification and production of Plasmodium falciparum zygotes from in vitro culture using magnetic column and Percoll density gradient
Source: Malar J. 2020 May 25;19:192. doi: 10.1186/s12936-020-03237-1 (PMC7249376; doi:10.1186/s12936-020-03237-1)
Supplement: Supplementary file 6 — Additional file 6: Table S4. Enrichment of zygotes during purification using “2 MACS 2 Percoll” method. [file 12936_2020_3237_MOESM6_ESM.pdf]

Table S4: Enrichment of zygotes during purification using "2 MACS 2 Percoll" method

Parasite/Cell	Before Purification (after 6h in ookinete media)	After 1st MACS Column	After 2nd MACS Column	After 1st Percoll Column		After 2nd Percoll Column		
				upper band	lower band	upper band	middle band	lower band
RBC	11.7x10^9	5.7x10^6	0	0	0	0	0	0
Gam	27.0x10^7	3.0x10^7	2.6x10^7	2.6x10^6	3.7x10^6	0	0	1.0x10^5
Zygote	11.6x10^7	3.5x10^7	3.2x10^7	1.6x10^7	2.3x10^6	6.4x10^6	4.7x10^5	3.0x10^5
Mac Gam	2.3x10^7	4.7x10^6	2.7x10^5	1.5x10^5	6.0x10^5	0	0	0
Zygote %	1.0%	46.4%	54.9%	85.3%	34.8%	100%	100.0%	75.0%

Absolute total numbers of RBCs and different parasite stages before and after each purification step are shown. Enrichment of zygote is indicated by the percentage of zygote in the whole population after each purification step. RBC = red blood cells; Gam = gametocytes; Mac Gam = macrogametocytes.
