# Supplementary material for: Purification and production of Plasmodium falciparum zygotes from in vitro culture using magnetic column and Percoll density gradient
Source: Malar J. 2020 May 25;19:192. doi: 10.1186/s12936-020-03237-1 (PMC7249376; doi:10.1186/s12936-020-03237-1)
Supplement: Supplementary file 7 — Additional file 7: Table S5. Transformation of zygotes into ookinetes after purification. [file 12936_2020_3237_MOESM7_ESM.pdf]

**Table S5: Transformation of zygotes into ookinetes after purification**

| Parasite/Cell | 1 MACS 1 Accudenz | 1 MACS 1 Percoll | | |
|---|---|---|---|---|
| | upper band | upper band | middle band | lower band |
| Zygote | 2.0x10^6 | 4.0x10^6 | 4.2x 10^6 | 2.1x10^6 |
| Ookinete | 1.1x10^5 | 2.2x10^5 | 1.2x10^5 | 1.1x10^5 |
| P trans (z-o) | 5.5% | 5.5% | 2.9% | 5.2% |

Absolute total number of zygotes after 1 MACS 1 Accudenz or 1 MACS 1 Percoll purification are shown. The number of ookinetes after transformation and percentage of zygotes that transformed into ookinetes are also given. P trans (z-o) = percent transformation, zygote to ookinete.
